# Enhanced VEGF production and decreased immunogenicity induced by TGF-**β** 1 promote liver metastasis of pancreatic cancer

**DOI:** 10.1054/bjoc.2001.1941

**Published:** 2001-08

**Authors:** H Teraoka, T Sawada, T Nishihara, M Yashiro, M Ohira, T Ishikawa, H Nishino, K Hirakawa

**Affiliations:** First Department of Surgery, Osaka City University Medical School, 1–4–3 Asahi-machi, Abeno-ku, Osaka, 545–8585, Japan

**Keywords:** TGF-β 1, liver metastasis, pancreatic cancer, VEGF

## Abstract

TGF-βs are multifunctional polypeptides that regulate cell growth and differentiation, extracellular matrix deposition, cellular adhesion properties, angiogenesis and immune functions. In this study, we investigated the effect of TGF-β1 on liver metastasis and its mechanism by using human pancreatic cancer cell lines Panc-1, Capan-2, and SW1990. Capan-2 and SW1990 cells demonstrated enhanced liver metastatic potential by in vivo splenic injection with TGF-β1. Consequently, we examined the role of TGF-β1 on in vitro angiogenesis and received cytotoxicity by peripheral blood mononuclear leukocytes (PBMLs). While TGF-β1 slightly decreased cell proliferation, it also upregulated VEGF production in all cancer cells examined. The binding of PBMLs to cancer cells and cancer cell cytotoxicity during co-culture with PBMLs were remarkably decreased by treatment with TGF-β1. Panc-1 cells revealed no liver metastasis despite their high immunogenetic and angiogenetic abilities, which was attributed to a lack of expression of the cell surface carbohydrates that induce attachment to endothelial cells. We concluded that the presence of TGF-β1 in the microenvironment of tumour site might play an important role in enhancing liver metastasis of pancreatic cancer by modulating the capacity of angiogenesis and immunogenicity. © 2001 Cancer Research Campaign http://www.bjcancer.com


*British Journal of Cancer* (2001) **85**(4), 612–617
© 2001 Cancer Research Campaign
doi: 10.1054/ bjoc.2001.1941, available online at http://www.idealibrary.com on IDEAL

http://www.bjcancer.com

### Enhanced VEGF production and decreased immunogenicity induced by TGF-β 1 promote liver metastasis of pancreatic cancer

**H Teraoka, T Sawada, T Nishihara, M Yashiro, M Ohira, T Ishikawa, H Nishino and K Hirakawa**

First Department of Surgery, Osaka City University Medical School, 1–4–3 Asahi-machi, Abeno-ku, Osaka 545–8585, Japan

**Summary** TGF-βs are multifunctional polypeptides that regulate cell growth and differentiation, extracellular matrix deposition, cellular adhesion properties, angiogenesis and immune functions. In this study, we investigated the effect of TGF-β1 on liver metastasis and its mechanism by using human pancreatic cancer cell lines Panc-1, Capan-2, and SW1990. Capan-2 and SW1990 cells demonstrated enhanced liver metastatic potential by in vivo splenic injection with TGF-β1. Consequently, we examined the role of TGF-β1 on in vitro angiogenesis and received cytotoxicity by peripheral blood mononuclear leukocytes (PBMLs). While TGF-β1 slightly decreased cell proliferation, it also upregulated VEGF production in all cancer cells examined. The binding of PBMLs to cancer cells and cancer cell cytotoxicity during co-culture with PBMLs were remarkably decreased by treatment with TGF-β1. Panc-1 cells revealed no liver metastasis despite their high immunogenetic and angiogenetic abilities, which was attributed to a lack of expression of the cell surface carbohydrates that induce attachment to endothelial cells. We concluded that the presence of TGF-β1 in the microenvironment of tumour site might play an important role in enhancing liver metastasis of pancreatic cancer by modulating the capacity of angiogenesis and immunogenicity. © 2001 Cancer Research Campaign http://www.bjcancer.com

**Keywords**: TGF-β 1, liver metastasis, pancreatic cancer, VEGF

Despite recent advances in diagnostic and surgical procedures, pancreatic cancer has one of the worst prognoses among cancers of the digestive organs because of its high rate of liver metastasis and local recurrence (Hiraoka, 1990). However, the cellular biology and molecular mechanism of liver metastasis remain poorly understood.

Transforming growth factor-βs (TGF-βs) are multifunctional polypeptides often characterized as negative regulators of epithelial cell growth. TGF-βs regulate not only cell growth but also differentiation, extracellular matrix deposition, cellular adhesion properties, angiogenesis and immune functions (Massague, 1990; Sporn and Roberts 1992). Several investigators have reported that TGF-β expression in a variety of malignancies has been associated with increased tumorigenesis, and transforming growth factor-β 1 (TGF-β1) overexpression has been correlated with disease progression in pancreatic, breast, pulmonary and gastric carcinomas, osteosarcomas and melanomas (Friess et al, 1993; Gorsch et al, 1992; Takanami et al, 1997; Nishimura et al, 1998; Kloen et al, 1997; Moretti et al, 1997). But the biological effect of TGF-β1 on liver metastasis and its mechanism in pancreatic cancer remain to be clarified.

The process of liver metastasis is known to consist of multiple steps, including detachment of malignant cells from the primary tumour, degradation of and invasion into the extracellular matrix by proteases, infiltration into blood vessels, adhesion to endothelial cells and extravasation at a distant site, and proliferation. Moreover, immunogenicity and angiogenesis are also thought to play an important role in determining the capacity of tumour invasion and metastasis.

The purpose of this study was to elucidate the effect of TGF-β1 on liver metastasis and its mechanism by examining the capacity of angiogenesis and immunogenicity using human pancreatic cancer cell lines.

### MATERIALS AND METHODS

### Mice

Balb/c nude mice (Oriental Kobo, Tokyo, Japan), 6-week-old female, were used in the experiments. All experiments with nude mice were performed by regarding their welfare in accordance with the guidelines approved by the United Kingdom Co-ordinating Committee on Cancer Research (UKCCCR).

### Cell lines and cell culture

Human pancreatic cancer cell lines Panc-1, Capan-2, and SW1990 were cultured in Dulbecco's modified Eagle's medium (DMEM; Bioproducts, Walkersville, MD) with 10% fetal calf serum (FCS; Gibco, Grand Island, NY), 100 IU/ml penicillin (ICN Biomedicals, Costa Mesa, CA), 100 IU/ml streptomycin (ICN Biomedicals) and sodium pyruvate (Bioproducts). Cultures were maintained at 37˚C in a humidified atmosphere of 5% $CO_2$.

### In vivo liver metastatic assay

Panc-1, Capan-2, and SW1990 cells were incubated in DMEM with either 10% fetal calf serum alone or 10% fetal calf serum and

*Received 18 September 2000*
*Revised 3 May 2001*
*Accepted 8 May 2001*

*Correspondence to:* H Teraoka

TGF-β 1 (20 ng/ml) for 48 h. The cells were then suspended to a final concentration of $5 \times 10^5$/0.1 ml in phosphate-buffered saline (PBS) and injected into the spleen of 6-week-old female Balb/c nude mice under ether anaesthesia. After injection, the spleen was extracted. The mice were sacrificed after 5 weeks to measure the number of metastatic tumour nodules and the liver weight.

### Measurement of vascular endothelial growth factor (VEGF) levels in culture supernatants

The cells were cultured in DMEM with 10% FCS, washed with DMEM, and then cultured in DMEM alone or with 0, 1, 10, or 20 ng/ml TGF-β 1 for 48 h. The culture supernatants were collected and their VEGF levels were measured using commercially available ELISA kits (Amersham International PLC, Buckinghamshire, England).

### Isolation and purification of peripheral blood mononuclear leukocytes (PBMLs) from healthy human donors

Human peripheral blood mononuclear leukocytes (PBMLs) were prepared from healthy volunteers by Ficoll-Hypaque density gradient centrifugation. Mono-Poly Resolving Medium was added to plastic non-siliconized tubes and then fresh anti-coagulated blood were added onto the medium. The tubes were centrifuged at 1500 rpm for 30 min, and the supernatant was drawn off with a pasteur pipette. After two washes with PBS, PBMLs were collected and resuspended in DMEM with 10% FCS. The resuspended lymphocytes were seeded onto a plastic plate and incubated for 1 h to remove attached monocytes. PBMLs were resuspended in medium appropriate to the work being done.

### Lymphocytes adhesion assay

The binding of PBMLs to cancer cells was also investigated. Panc-1, Capan-2, and SW1990 cells ($2.5 \times 10^5$/well) were incubated in DMEM with either 10% fetal calf serum alone or 10% fetal calf serum and TGF-β 1(20 ng/ml) for 48 h in 96-well microtitre plates. After washing these plates with PBS, PBMLs ($2.5 \times 10^6$/well) suspended in serum-free medium were allowed to attach to cancer cells on each well for 1 h at 37˚C. The binding of PBMLs was quantified by measuring the concentration of 3-(4,5-dimethylthiazol-2yl)-2,5-dipheny l-2,4-tetrazolium bromide (MTT; Sigma Chemical Co., St Louis, MO) by colorimetric assay (Carmichael et al, 1987), using a MTP-120 Microplate reader at 550 nm. The percentage of total PBMLs that adhered to cancer cells (% of adhesion) was calculated as

% of adhesion = (OD of experimental wells – OD of cancer cell wells) / OD of total PBML wells × 100.

### Cancer cell cytotoxicity assay

The cell cytotoxicity assay was performed using a Cytotox 96 Non Radioactive Cytotoxicity Assay Kit (Promega Co., Madison, WI), that measured lactate dehydrogenase(LDH), a stable cytosolic enzyme released upon cell lysis during culturing of mixed leukocytes (effector cells) and tumour cells (target cells) culture (MLTC). Effector cells (PBMLs) were isolated from healthy donors by Ficoll-Hypaque density gradient centrifugation as described above. After target cells were pretreated with or without 20 ng/ml TGF-β 1 for 48 h prior to assay, approximately $1\times 10^4$ target cells were placed in each well of a 96-well plate, and then $1\times 10^5$ effector cells were added and incubated at 37˚C in a humidified atmosphere of 5% $CO_2$ for 18 h. After the addition of Stop Solution (1 M acetic acid), absorbance related to released LDH level in the medium was measured with an MTP-120 Microplate reader (Corana Electric Co., Ibaragi, Japan) at 492 nm. Then the percentage of total cancer cells that underwent lysis (% cytotoxicity) was calculated as

% cytotoxicity = (Experimental – Effector spontaneous – Target Spontaneous) / (Target maximum – Target Spontaneous) × 100.

### Enzyme-linked immunosorbent assay (ELISA)

To determine the effect of TGF-β 1 on cell surface antigen expression, cancer cells were cultured in 96-well microtitre plates (Coster, Cambridge, MA) for 48 h in the presence or absence of TGF-β 1(20 ng/ml). After the cells had been washed three times with PBS, they were fixed with 0.25% glutaraldehyde at room temperature for 1 h. After blocking the wells with 100 mM glycine/1%BSA in PBS, anti-sialyl $Le^a$ and $Le^x$ first antibody were reacted to cells and ELISAs were performed with horseradish peroxidase-conjugated second antibodies (Zymed Laboratories Inc., San Francisco, CA) as previously described (Sawada et al, 1994; Ho et al, 1993).

### Cell proliferation assay

To examine the effect of TGF-β 1 on cell proliferation, $1 \times 10^4$ cancer cells with or without TGF-β 1 were inoculated onto a 24-well culture plate (Falcon, Lincoln Park, NJ). After 24, 48, and 72 h of incubation, cells were harvested and cell numbers were counted using a Coulter Counter (Coulter Electronics, Luton, UK)

### Statistical analysis

Results were expressed as the mean ± SD. Student's *t*-test was used for statistical analysis, and *P* values less than 0.05 were considered to indicate statistical significance.

### RESULTS

### Effect of TGF-β1 on results of liver metastasic assay

Using a splenic injection model in nude mice, we investigated whether in vivo liver metastasis of Panc-1, Capan-2 and SW1990 cells was enhanced by TGF-β1 pretreatment. Macroscopic and histological examination disclosed low metastasis in untreated Panc-1 and Capan-2 cells (0% and 18.2%), while untreated SW1990 cells induced liver metastasis in 9 of 10 mice (90%). After pretreatment with TGF-β1, both Capan-2 and SW1990 cells enhanced liver metastatic potential (by 84.6% and 100%, respectively), but Panc-1 cells did not induce any liver metastasis (Figure 1, Table 1).

### Effect of TGF-β1 on VEGF production

The concentrations of VEGF in the culture supernatant were 0.91 ± 0.04, 1.01 ± 0.08, and 1.60 ± 0.08 ng/ml for untreated Panc-1,

© 2001 Cancer Research Campaign 

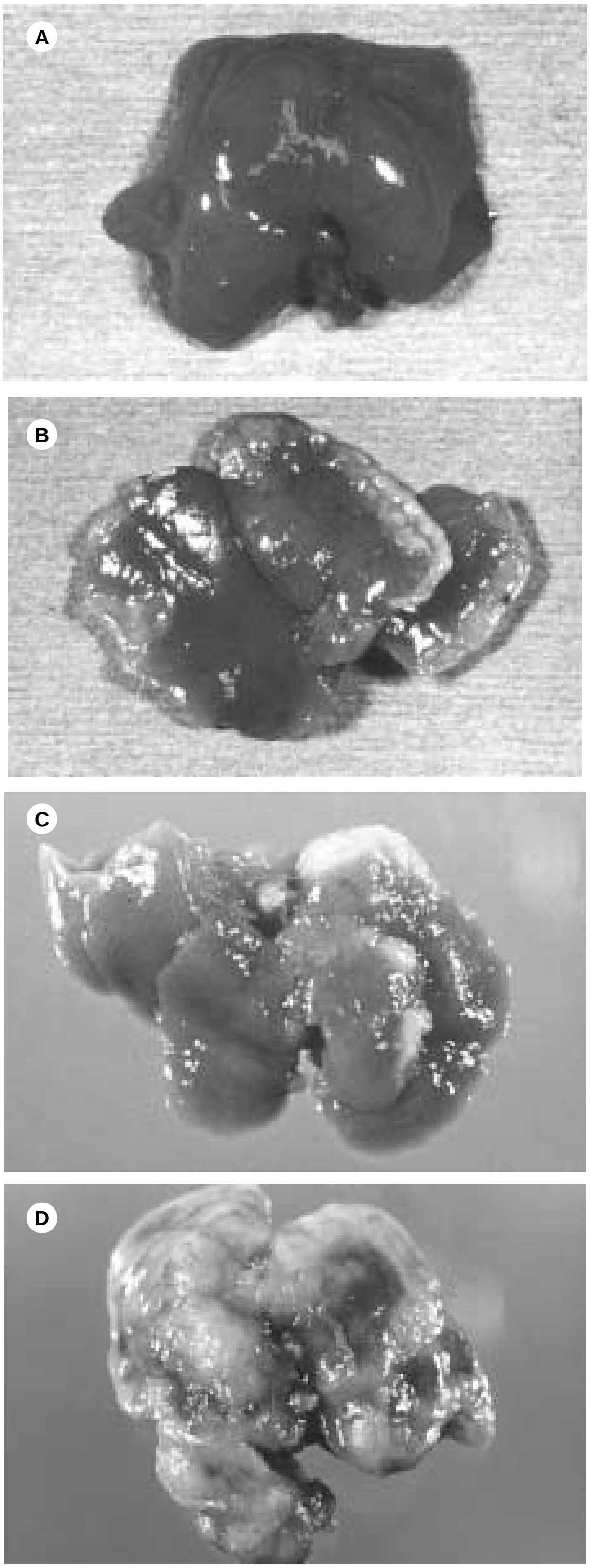

**Figure 1** Macroscopic findings of the resected liver in nude mice administered splenic injection of cancer cells. Capan-2 cells originally revealed no liver metastasis (**A**) but demonstrated high incidence of liver metastasis by pretreatment with TGF-β 1(20 ng/ml) for 48 h before injection (**B**). SW1990 cells also showed enhanced liver metastasis following TGF-β1 (20 ng/ml) treatment (**D**), compared with untreated cells (**C**)

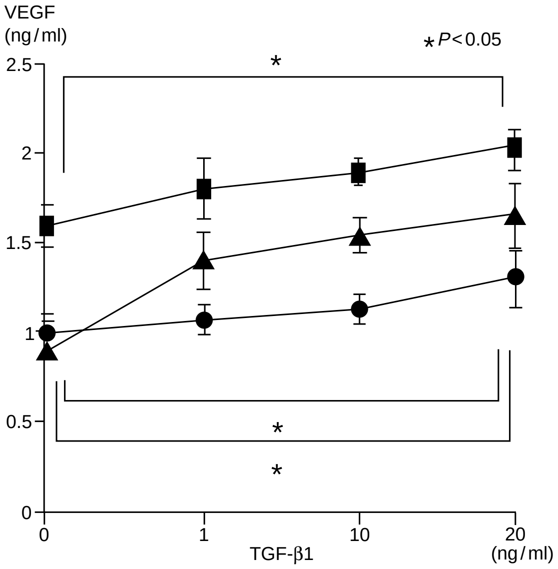

**Figure 2** Effect of TGF-β1 on VEGF production in the culture supernatant of SW1990 (■), Capan-2 (●), and Panc-1 (▲) cells. Cells were treated every 48 h. After the addition of TGF-β1 (from 0 to 20 ng/ml), VEGF levels in the culture supernatant were significantly increased in all three cancer cell lines in a dose-dependent manner. Results are means ± SD of four samples

Capan-2, and SW1990 cells, respectively. The highest production level was observed in highly metastatic SW1990 cells. After the addition of TGF-β1 (from 0 to 20 ng/ml), VEGF levels in the culture supernatant were significantly increased in all three cancer cell lines in a dose-dependent manner. After treatment with TGF-β1 (20 ng/ml), VEGF concentration in the culture supernatant were 1.65 ± 0.21, 1.31 ± 0.20, and 1.89 ± 0.13 ng/ml for Panc-1, Capan-2, and SW1990 cells, respectively (Figure 2).

### Effect of TGF-β1 on adhesion of lymphocytes to cancer cells

The attachment of PBMLs to cancer cells was inhibited following the addition of TGF-β 1 at a concentration of 20 ng/ml, compared with untreated cancer cells. The percentages of PBMLs binding to Panc-1, Capan-2 and SW1990 cells following the addition of TGF-β 1 were 24.3 ± 4.7%, 36.7 ± 10.8% and 11.2 ± 6.0%, while those of the controls were 34.5 ± 10.0, 59.5 ± 8.7% and 19.4 ± 6.6% (Figure 3).

### Effect of TGF-β1 on cancer cell cytotoxicity by PBMLs

The percentages of total Panc-1, Capan-2 and SW1990 cells showing cytotoxicity during 18-h co-culture with PBMLs were 43.5 ± 4.0%, 63.9 ± 10.5% and 30.2 ± 7.7%, respectively; however, after pretreatment with TGF-β1 at a concentration of 20 ng/ml, these percentages were remarkably decreased to 29.5 ± 5.6%, 35.5 ± 5.3% and 21.0 ± 5.6% (Figure 4).

### Effect of TGF-β1 on cell surface antigen expression

Expression of the cell surface antigen of sialyl Lewis-A ($sLe^a$) was higher in SW1990 than Capan-2 cells. In contrast, sialyl Lewis-X ($sLe^x$) was only detected in SW1990 cells. Panc-1 cells did not express either $sLe^a$ or $sLe^x$ antigens. TGF-β 1 had no influence on either $sLe^a$ or $sLe^x$ expression (Figure 5).

© 2001 Cancer Research Campaign

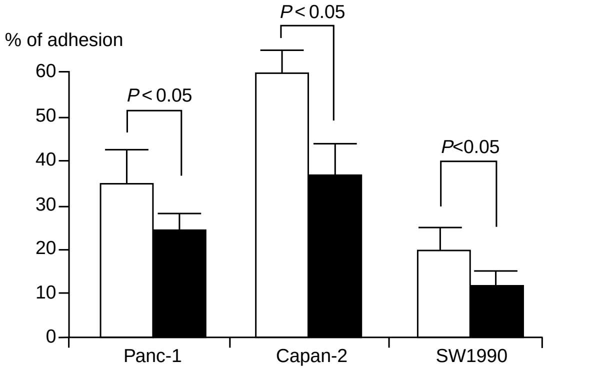

**Figure 3** Effect of TGF-β 1 on adhesion of lymphocytes to cancer cells. Panc-1, Capan-2 and SW1990 cells were incubated with (■) or without (❑) TGF-β 1 (20 ng/ml) for 48 h, and then PBMLs were allowed to attach to each cancer cell line for 1 h. The attachment of PBMLs to cancer cells was inhibited by pretreatment of TGF-β 1 in all three cells lines. Results are means ± SD of six independent experiments

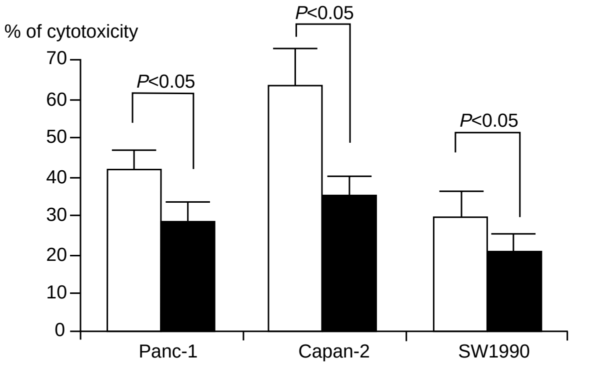

**Figure 4** Effect of TGF-β 1 on cancer cell cytotoxicity by PBMLs. Panc-1, Capan-2 and SW1990 cells with (■) or without (❑) TGF-β 1 (20 ng/ml) were co-cultured with PBMLs for 18 h. The cytotoxicities of Panc-1, Capan-2 and SW1990 cells were remarkably decreased after pretreatment with TGF-β 1. Results are means ± SD of six independent experiments

### Effect of TGF-β 1 on cell proliferation

At a concentration of 20 ng/ml, the addition of TGF-β 1 slightly reduced the proliferation of all cells, but there were no significant difference in proliferation among the cell lines, even at 72 h of incubation (data not shown).

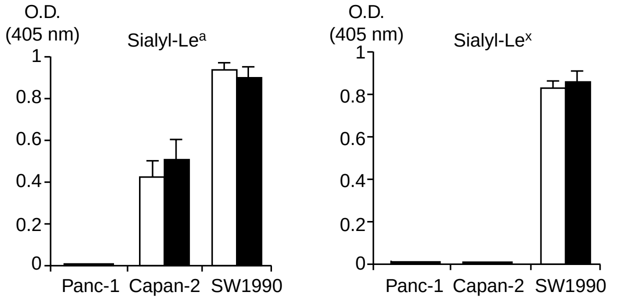

**Figure 5** Effect of TGF-β 1 on cell surface antigen expression by ELISA. SW1990, Panc-1 and Capan-2 cells were cultured in the presence (■) or absence (❑) of TGF-β 1 (20 ng/ml) for 48 h. $sLe^a$ expression on SW1990 and Capan-2, and $sLe^x$ expression on SW1990 were not affected by TGF-β 1 treatment. Panc-1 did not reveal the expression of $sLe^a$ and $sLe^x$

### DISCUSSION

In the present study, we found that TGF-β1 enhanced the liver metastasis of pancreatic cancer by modulating the capacity for immunogenicity and angiogenesis.

Haematogenous metastasis involves a multistep process that begins with detachment of tumour cells from the primary lesion and ends with their attachment to a different organ and formation of new tumour nodules (Fidler, 1995). In these steps, tumour growth depends on angiogenesis to a large degree, which means that the tumours are dependent on the ingrowth of a vascular supply from the surrounding tissue in order to proliferate and metastasize (Folkman, 1995; McCulloch et al, 1995). Any individual tumour may have dominant angiogenic factors that induce angiogenesis by favouring an imbalance between positive and negative regulators. These angiogenic regulators act either directly on endothelial cells or indirectly by inducing the production of other regulators. Among these factors, VEGF is particularly important, since it clearly acts on endothelial cells in a direct manner (Mattern et al, 1997). Recently, it has been reported that enhanced VEGF expression correlates with haematogenous metastasis and prognosis in human colon, gastric, breast and pancreatic cancers (Kang et al, 1997; Maeda et al, 1996; Toi et al, 1994; Ikeda et al, 1999). These findings support our results that the production of VEGF in the culture supernatant of SW1990 was higher than that in the other two cell lines, and that only SW1990 originally revealed liver metastatic potential by in vivo splenic injection.

The production of many angiogenic modulators, such as VEGF, is regulated by various factors. It has been demonstrated that TGF-β1 has an indirect effect on angiogenesis. TGF-β1 promotes blood vessel

**Table 1** Effect of TGF-β 1 on liver metastasis

| | | Liver metastasis in nude mice (%) | Number of liver metastases | Liver weight (g) |
|---|---|---|---|---|
| Panc-1 | Control | 0 (0/10)[b] | 0 | 1.7 ± 0.4 |
| | TGF-β1 treatment group | 0 (0/10) | 0 | 1.6 ± 0.4 |
| Capan-2 | Control | 18.2 (2/11)[a] | 0.4 ± 1.6[a] | 1.4 ± 0.2 |
| | TGF-β1 treatment group | 84.6 (11/13)[a] | 8.2 ± 7.1[a] | 1.6 ± 0.3 |
| SW1990 | Control | 90 (9/10) | Innumerable | 2.9 ± 0.9[a] |
| | TGF-β1 treatment group | 100 (10/10) | Innumerable | 4.0 ± 1.01[a] |

Data shown are the mean ± SD. TGF-β 1: transforming growth factor-beta 1. [a]: $P < 0.01$. [b]: the number of nude mice having metastatic nodules in the liver / total number of nude mice.

© 2001 Cancer Research Campaign formation by potentiating VEGF- and bFGF-dependent angiogenesis in vascular smooth muscle cells (Brogi et al, 1994). It also upregulates the production of numerous proangiogenic factors, including VEGF, bFGF, platelet-derived growth factor, tumor necrosis factor-α, and interleukin-1 (Pepper, 1997). Our results showed that TGF-β1 up regulates the production of VEGF in all three pancreatic cancer cell lines examined. These findings suggested that the presence of TGF-β1 in the microenvironment produced by tumours and their surrounding tissues may play an important role in enhancing the liver metastasis of pancreatic cancer by modulating the capacity of angiogenesis with up-regulation of VEGF production.

Pertovaara et al (1994) reported that TGF-β treatment of human lung adenocarcinoma A549 cells results in the induction of VEGF mRNA. They also found that TGF-β induced the expression of c-jun, junB, and c-fos genes and the activation of AP-1 transcription factors in A549 cells (Pertovaara et al, 1989). On the other hand, it has been shown that the promoter region of the VEGF gene contains several potential binding sites for AP-1 (Tischer et al, 1991). Thus these transcription factors could mediate VEGF induction by TGF- β.

TGF-β1 has also been shown to promote angiogenesis directly. Some of the most compelling evidence comes from TGF-β1 null mice, in which homozygous deletion of TGF-β1 is lethal. These mice have significant defects in the yolk sac vasculature and hematopoietic system, including increased vessel wall fragility and frequent disruptions between endothelial cells. This demonstrates that a deficiency in the production of TGF-β1 markedly affects the establishment and maintenance of vessel wall integrity (Dickson et al, 1995).

Among its numerous functions, TGF-β1 has been shown to act as a potent immunosuppressive factor by inhibiting the proliferation and function of natural killer (NK) cells, lymphokine-activated killer (LAK) cells, cytotoxic T-cells and B-cells (Kehrl et al, 1986; Tada et al, 1991; Rook et al, 1986; Espevik et al, 1988; Wahl et al, 1989). Malignant tumour cells escape from these effector cells and then can grow and metastasize. Immunogenicity is thus also thought to be important in determining the capacity for tumour invasion and metastasis.

Cytotoxic lymphocytes adhere to cancer cells and lyse them by recognizing various molecules on their surface. Among the molecules, HLA class I and II antigens, ICAM-1, B 7, and LFA-3 have been reported to play important roles in mediating the cytotoxic effects of lymphocytes (Ishii et al, 1994; Damale, 1992). In the present study, we found that TGF-β1 inhibit the attachment of PBML to cancer cells and decreased cell cytotoxicity, both of which may increase the capacity for metastasis. These results suggested that TGF-β1 may influence the surface expression of these adhesion molecules. In the future, the detailed mechanisms of this phenomenon should be investigated.

There are many other adhesion molecules on the surface of cancer cells, such as the carbohydrate antigens, $sLe^a$ and $sLe^x$, both of which are known to have a strong connection with hematogenous metastasis in pancreatic cancer (Sawada et al, 1994; Kishimoto et al, 1996). These antigens are important ligands for E-selectin, which mediates the attachment between cancer cells and endothelial cells in target organs in the cancer cell lines of several species (Takada et al, 1991; Iwai et al, 1993). Okazaki et al (1998) also reported that $sLe^a$ and $sLe^x$ appear to be involved in the increase of metastatic activity of colon cancer. The present finding that $sLe^a$ and $sLe^x$ were highly expressed in highly metastatic SW1990 cells was consistent with these previous reports. We then investigated the effect of TGF-β1 on the cell surface expression of $sLe^a$ and $sLe^x$, but found that it had no influence. Our present results showed that the liver metastatic potential of Panc-1 cells was not enhanced by in vivo splenic injection after the treatment of TGF-β1, although the capacity for immunogenicity and angiogenesis were modulated by TGF-β1 as in the other two cancer cell lines. Panc-1 cells expressed neither $sLe^a$ nor $sLe^x$, while Capan-2 cells expressed $sLe^a$. Therefore Panc-1 cells must be lacking in adhering to endothelial cells, an important process in the development of hematogenous metastasis. This is supposed to be the reason why Panc-1 cells did not metastasize after treatment of TGF-β1, compared to Capan-2 cells.

TGF-β1 is known to be potent inhibitor of proliferation in most cells, including some cancer cells, and exerts its effects through an interaction with type I and type II membrane receptors. Recently, it has been demonstrated that a loss of responsiveness to TGF-β-mediated growth inhibition was involved in alteration of these receptors and in tumour progression (Kim et al, 1998). In the present study, TGF-β1 only slightly affected the proliferation of cancer cells but significantly enhanced liver metastasis. These results might be related to the alteration of TGF-β receptors and / or to the alteration of postreceptors that are secondary to structural or functional abnormalities in the p53 or DPC4 tumour suppressor genes (Wyllie et al, 1991; Hahn et al, 1996).

In summary, we conclude that the presence of TGF-β1 in the microenvironment of the tumour site may play an important role in enhancing the liver metastasis of pancreatic cancer by promoting the escape of cancer cells from immunosurveillance, in addition to its effect on tumour angiogenesis. Further elucidation of this process might lead to development of new therapeutic strategies and, in turn, to a decrease in the high morbidity and mortality of pancreatic cancer.

### ACKNOWLEDGEMENTS

This study was supported in part by a Grant-in-Aid for Scientific Research from the Ministry of Education, Science, Sports and Culture of Japan.

### REFERENCES

Brogi E, Wu T, Namiki A and Isner JM (1994) Indirect angiogenic cytokines upregulate VEGF and bFGF gene expression in vascular smooth muscle cells. *Circulation* **90**: 649–652

Carmichael J, DeGraff WG, Gazdar AF, Minna JD and Mitchell JB (1987) Evaluation of a tetrazolium-based semiautomated colorimetric assay: assessment of chemosensitivity testing. *Cancer Res* **47**: 936–942

Damale NK (1992) Differential costimulatory effects of adhesion molecules B 7, ICAM-1, LFA-3, and VCAM-1 on resting and antigen-primed $CD4^+$ T lymphocytes. *J Immunol* **148**: 1985–1992

Dickson MC, Martin JS, Cousins FM, Kulkarni AB, Karlsson S and Arkhurst RJ (1995) Defective haemetaopoiesis and vasculogenesis in transforming growth factor-β 1 knockout mice. *Development* **121**: 1845–1854

Espevik T, Figari IS, Ranges GE and Palladino MA (1988) Transforming growth factor-β1 (TGF-β1) and recombinant human tumor necrosis factor-α reciprocally regulate the generation of lymphokine-activated killer cell activity. *J Immunol* **140**: 2312–2316

Fidler IJ (1995) Modulation of the organ microenvironment for treatment of cancer metastasis. *J Natl Cancer Inst* **21**: 1588–1592

Folkman J (1995) Clinical applications of research on angiogenesis. *N Engl J Med* **333**: 1757–1763

Friess H, Yamanaka Y, Buchler M, Ebert M, Beger HG, Gold LI and Korc M (1993) Enhanced expression of transforming growth factor-β isoforms in pancreatic cancer correlates with decreased survival. *Gastroenterology* **105**: 1846–1856

*© 2001 Cancer Research Campaign*

Gorsch SM, Memoli VA, Stukel TA, Gold LI and Arrick BA (1992) Immnohistochemical staining for transforming growth factor-beta 1 associates with disease progression in human breast cancer. *Cancer Res* **52**: 6949–6952

Hahn SA, Schutte M, Hoque AT, Moskaluk CA, da Costa LT, Rozenblum E, Weinstein CL, Fischer A, Yeo CJ, Hruban RH and Kern SE (1996) DPC4, a candidate tumor suppressor gene at human chromosome 18q21.1. *Science* **271**: 350–353

Hiraoka T (1990) Extended radical resection of cancer of the pancreas with intraoperative radiotherapy. *Clin Gastroenterol* **4**: 985–993

Ho JJL, Bi N, Siddiki B, Chung Y-S, Yuan M and Kim YS (1993) Multiple forms of intracellular and secreted mucins in a pancreatic cancer cell line. *Cancer Res* **53**: 884–890

Ikeda N, Adachi M, Taki T, Huang C, Hashida H, Takabayashi A, Sho M, Nakajima Y, Kanehiro H, Hisanaga M, Nakano H and Miyake M (1999) Prognostic significance of angiogenesis in human pancreatic cancer. *Br J Cancer* **79**: 1553–1563

Ishii H, Gouchi A and Orita K (1994) The enhancement of cell surface ICAM-1 and HLA Class antigens in human gastric cancer cell lines by IFN-gamma. *Acta Med Okayama* **48**: 73–79

Iwai K, Ishikura H, Kaji M, Sugiura H, Ishizu A, Takahashi C, Kato H, Tanabe T and Yoshiki T (1993) Importance of E-selectin(ELAM-1) and sialyl Lewisa in the adhesion of pancreas carcinoma cells to activated endothelium. *Int J Cancer* **54**: 972–977

Kang SM, Maeda K, Chung YS and Sowa M (1997) Vascular endothelial growth factor expression correlates with hematogenous metastasis and prognosis in colorectal carcinoma. *Oncol Reports* **4**: 381–384

Kehrl JH, Wakefield LM, Roberts AB, Jakowlew S, Alvarez-Mon M, Derynck R, Sporn MB and Fauci AS (1986a) Production of transforming growth factor β by human T lymphocytes and its potential role in the regulation of T cell growth. *J Exp Med* **163**: 1037–1050

Kehrl JH, Roberts AB, Wakefield LM, Jakowlew S, Sporn MB and Fauci AS (1986b) Transforming growth factor β is an important immunomodulatory protein for human B lymphocytes. *J Immonol* **137**: 3855–3860

Kim IY, Ahn HJ, Lang S, Oefelein MG, Oyasu R, Kozlowski JM and Lee C (1998) Loss of expression of transforming growth factor-β receptors is associated with poor prognosis in prostate cancer patients. *Clin Cancer Res* **4**: 1625–1630

Kishimoto T, Ishikura H, Kimura C, Takahashi T, Kato H and Yoshiki T (1996) Phenotypes correlating to metastatic properties of pancreas adenocarcinoma in vivo: the importance of surface sialyl Lewisa antigen. *Int J Cancer* **69**: 290–294

Kloen P, Gebhardt MC, Perez-Atay de A, Rosenberg AE, Springfield DS, Gold LI and Mankin HJ (1997) Expression of transforming growth factor-beta (TGF-beta) isoforms in osteosarcomas: TGF-beta 3 is related to disease progression. *Cancer* **80**: 2230–2239

Maeda K, Hirakawa K, Ogawa Y, Takatsuka S, Kang SM, Ogawa M, Sawada T and Sowa M (1996) Prognostic value of vascular endothelial growth factor expression in gastric carcinoma. *Cancer* **77**: 858–863

Massague J (1990) The transforming growth factor-beta family. *Ann Rev Cell Biol* **6**: 597–641

Mattern J, Koomagi R and Volm N (1997) Coexpression of VEGF and bFGF in human epidermoid lung cancer associated with increased vessel density. *Anticancer Res* **17**: 2249–2252

McCulloch P, Choy A and Martin L (1995) Association between tumor angiogenesis and tumor cell shedding into effluent venous blood during breast cancer surgery. *Lancet* **346**: 1334–1335

Moretti S, Pinzi C, Berti E, Spallanzani A, Chiarugi A, Boddi V, Reali UM and Giannotti B (1997) In situ expression of transforming growth factor-beta is associated with melanoma progression and correlates with Ki67, HLA-DR and beta 3 integrin expression. *Melanoma Res* **7**: 313–321

Nishimura S, Hirakawa K, Yashiro M, Inoue T, Matsuoka T, Fujihara T, Murahashi K, Sawada T, Nakata B, Jikihara I, Takagi H and Sowa M (1998) TGF-beta 1 produced by gastric cancer cells affects mesothelial cell morphology in peritoneal dissemination. *Int J Oncology* **12**: 847–851

Okazaki K, Nakayama Y, Shibao K, Hirata K, Nagata N and Itoh H (1998) Enhancement of metastatic activity of colon cancer as influenced by expression of cell surface antigens. *J Surg Res* **78**: 78–84

Pepper MS (1997) Transforming growth factot-beta; vasculogenesis, angiogenesis and vessel wall integrity. *Cytokine Growth Factor Rev* **8**: 21–43

Pertovaara L, Sistonen L, Bos TJ, Vogt PK, Keski-Oja J and Alitalo K (1989) Enhanced *jun* gene expression is an early genomic response to transforming growth factor β stimulation. *Mol Cell Biol* **9**: 1255–1262

Pertovaara L, Kaipainen A, Mustonen T, Orpana A, Ferrara N, Saksela O and Alitalo K (1994) Vascular endothelial growth factor is induced in response to transforming growth factor-β in fibroblastic and epithelial cells. *J Biol Chem* **269**: 6271–6274

Rook AH, Kehrl, Wakefield LM, Roberts AB, Sporn MB, Burlington DB, Lane HC and Fauci AS (1986) Effects of transforming growth factor β on the functions of natural killer cells; Depressed cytolytic activity and blunting of interferon responsiveness. *J Immunol* **136**: 3916–3920

Sawada T, Ho JJL, Chung Y-S, Sowa M and Kim YS (1994) E-selectin binding by pancreatic tumor cells is inhibited by cancer sera. *Int J Cancer* **57**: 901–907

Sporn MB and Roberts AB (1992) The transforming growth factor-β: recent progress and new challenges. *J Cell Biol* **119**: 1017–1021

Tada T, Ohzeki S, Utsumi K, Takiuchi H, Muramatsu M, Li X, Shimizu J, Fujiwara H and Hamaoka T (1991) Transforming growth factor-β-induced inhibition of T cell function. *J Immunol* **146**: 1077–1082

Takada A, Ohmori K, Takahashi N, Tsuyuoka K, Yago A, Zenita K, Hasegawa A and Kannagi R (1991) Adhesion of human colon cancer cells to vascular endothelium mediated by a carbohydrate antigen, sialyl Lewisa. *Biochem Biophys Res Commun* **179**: 713–719

Takanami I, Tanaka F, Hashizume T, Kikuchi K, Yamamoto Y, Yamamoto T and Kodaira S (1997) Transforming growth factor-beta isoforms expression in pulmonary adenocarcinomas as prognostic markers: an immunohistological study of one hundred and twenty patients. *Oncology* **54**: 122–128

Tischer E, Mitchell R, Hartman T, Silva M, Gosp odarowicz D, Fiddes JC and Abraham JA (1991) The human gene for vascular endothelial growth factor. *J Biol Chem* **266**: 11947–11954

Toi M, Hoshina S, Takayanagi T and Tominaga T (1994) Association of vascular endothelial growth factor expression with tumor angiogenesis and with early relapse in primary breast cancer. *Jpn J Cancer Res* **85**: 1045–1049

Wahl SM, McCartney-Francis N and Mehrenhagen S (1989) Inflammatory and immunomodulatory roles of TGF-β. *Immunol Today* **10**: 258–262

Wyllie FS, Dawson T, Bond JA, Goretzki P, Game S, Prime S and Wynford-Thomas D (1991) Correlated abnormalities of transforming growth factor-β1 response and p53 expression in thyroid epithelial cell trasformation. *Mol Cell Endocrinol* **76**: 13–21

*© 2001 Cancer Research Campaign* 
